# FDA-Listed Interactive Devices for Home Movement Rehabilitation After Stroke: A Mixed-Methods Study of Availability, User Needs, Information Gaps, and an Accompanying Dataset

**DOI:** 10.3390/bioengineering13040387

**Published:** 2026-03-27

**Authors:** Luis Garcia-Fernandez, Juan C. Perez-Ibarra, Andria J. Farrens, Vicky Chan, Joshua J. Macopson, David J. Reinkensmeyer

**Affiliations:** 1 Mechanical and Aerospace Engineering Department, University of California Irvine, Irvine, CA 92617, USA; jperezib@hs.uci.edu (J.C.P.-I.); farrensa@uci.edu (A.J.F.); vchan2@hs.uci.edu (V.C.); joshuagj@hs.uci.edu (J.J.M.); dreinken@uci.edu (D.J.R.); 2 Department of Orthopaedics & Rehabilitation, University of New Mexico, Albuquerque, NM 87106, USA

**Keywords:** stroke, home-based rehabilitation, neurorehabilitation technology dataset, FDA-listed rehabilitation devices, user-centered decision support

## Abstract

Technologies for home movement rehabilitation after stroke are rapidly expanding. However, for consumers, the number and nature of available products are unclear, and the information provided by device manufacturers varies widely. To understand this landscape, we conducted a mixed-methods, descriptive study in which we used the U.S. Food and Drug Administration (FDA) database to identify interactive devices for stroke rehabilitation suitable for home use. We then surveyed 13 individuals with stroke to determine what information they most wanted about home-based rehabilitation devices and contacted manufacturers to obtain those details. Thirteen FDA codes were associated with stroke rehabilitation devices, encompassing 57 devices produced by 40 companies. Nearly half were categorized under two codes: QKC (interactive rehabilitation exercise devices) and GZI (neuromuscular stimulators). Among devices for which information was available, 71% were listed after 2015, and 23% cost under $1000. The top information priorities for individuals with stroke were required usage to achieve therapeutic benefit, expected benefit, ease of use, and motivational features. Despite repeated outreach, only 45% of companies responded to our queries; among those that did, details were vague and variable. These results confirm that a large and growing number of FDA-listed devices are now available for home-based post-stroke motor rehabilitation. We further identify a need to establish industry standards for reporting ease of use, motivational effectiveness, and dose–response characteristics to help the intended consumers select appropriate technologies. The curated dataset generated in this study is provided as a resource for future work and may support the development of accurate Artificial Intelligence-based interfaces for identifying and comparing rehabilitation devices.

## 1. Introduction

Stroke is a leading cause of major disability in the U.S. [1], with more than 600,000 people surviving a stroke each year, and over 9,000,000 individuals alive today who have experienced at least one stroke, 3% of the U.S. population [2,3,4]. Approximately 50% of chronic stroke survivors have hemiparesis, ~30% are unable to walk unassisted and ~30% have severe to moderate dependency in activities of daily living (Barthel Score < 60) [5]. Thus, persistent movement-related disability following stroke is a significant health care problem in the U.S.

Individuals typically receive hands-on therapy for several months after stroke to treat motor impairment and improve independence. Based on systematic reviews of dozens of trials, an intensity-effect relationship has been implicated between the amount of therapy individuals receive and the movement gains achieved [6,7,8,9,10,11]. However, one recent attempt to more precisely define this intensity-effect relationship for upper extremity therapy failed [12]. This failure may be due to the relatively small range of intensities tested (a range of 3200–9600 practice movements), highlighting the need to develop approaches for delivering motor training at higher dosage. As a point of reference, consider what is required in another motor learning context—learning to walk: toddlers take over 5 million steps in a year of learning to walk [13]. Recent studies that delivered higher doses of movement practice have found greater improvements in movement ability [14,15,16,17].

To become routinely incorporated into rehabilitation practice, such high-dose movement training will likely need to be semi-automated with technology. The amount of therapy a patient receives involving direct contact with rehabilitation therapists is often limited by cost considerations [18]. For example, the average length of stay for stroke survivors in inpatient rehabilitation facilities in the U.S. decreased approximately 54% (31.3 days to 14.5 days) after prospective payment system reimbursement was instituted in 1983 [19]. Even with a therapist, rehabilitation exercise is often not very intense [20]. Insurance companies will pay for a small and inconsistent number of therapy sessions [21]. Patients may exercise apart from a therapist; however, independent movement practice is particularly difficult for individuals who have movement impairment, which likely contributes to the reported poor compliance with home exercise programs [22,23,24,25]. Therefore, it is essential to develop motivating technologies for delivering effective forms of therapy at a manageable cost, so that individuals can exercise for longer periods and maximize recovery.

Rehabilitation devices that can be used semi-autonomously offer the potential for increased accessibility, more intensive practice, and greater independence during recovery. Recognizing this need, there has been a surge in the number of research groups and companies that are developing robotic, sensor, stimulation and vision-based devices for assisting in the movement rehabilitation of persons with disabilities, for the arms, hands, and legs (see reviews [26,27,28,29,30,31,32,33,34,35,36]). Most of this work has focused on the rehabilitation of movement after stroke because survivors of stroke are the largest target population, although there is also work on robotic movement training after spinal cord injury, cerebral palsy, and multiple sclerosis. Developers often state three main goals for this activity: automating the repetitive and/or strenuous aspects of therapy, delivering therapy in a more standardized manner, and quantifying outcomes with greater precision.

Despite the growing availability of such devices, stroke survivors and clinicians face challenges in selecting appropriate products. A few online directories exist, including the *NeuroRehab Directory* (a curated list of neurorehabilitation products), *MedicalExpo*’s rehabilitation systems section (a commercial listing of rehabilitation devices), and the *Exoskeleton Report catalog* (a registry of wearable exoskeletons). However, these resources are limited to specific device categories, do not systematically report clinical evidence, and/or are not updated in a way that ensures comprehensive coverage. As a result, potential users become aware of devices through internet searches, social networks, advertisements, word-of-mouth, and vendor displays at conferences. Further, the information available online about each device varies, and it is unclear whether this information matches well with the questions stroke survivors have about the devices. Even when trained rehabilitation therapists select devices, the devices they purchase often see limited uptake into regular clinical practice [37,38]. To address these challenges, we are developing a large-language-model-based chatbot to help stroke survivors and clinicians more easily search for and compare rehabilitation technologies [39]. To ensure accurate responses, the system will rely on a curated knowledge base containing verified information about available devices and their key characteristics.

Thus, the goal of this work was to develop a curated dataset of FDA-listed interactive rehabilitation devices suitable for home use after stroke. This dataset is provided in the Supplementary Materials and will be publicly accessible upon publication of this open-access article. This study presents (1) an analysis of the characteristics of the devices; (2) results from a survey of individuals post-stroke about the types of information they most want to know about the devices; and (3) insights gained from manufacturer responses to user-centered inquiries. Our findings aim to inform people post-stroke, caregivers, and therapists seeking effective home rehabilitation options. In addition, we seek to highlight areas where developers could improve information transparency and device design. Preliminary aspects of this work were presented previously in conference paper format [39] including interview-based survey development, initial identification of relevant FDA product codes, a preliminary dataset structure, first-round manufacturer outreach, and early results based on a limited sample. The present manuscript refines and extends that work by applying more stringent inclusion criteria, expanding the search and synthesis of publicly available online information about each device, revising and finalizing the dataset structure, publicly releasing the full dataset as Supplementary Materials, classifying devices into eight distinct technology categories, conducting additional manufacturer outreach, and reporting updated survey and manufacturer results based on a larger cohort, along with a substantially expanded analysis and discussion.

## 2. Materials and Methods

This work employed a mixed-methods, descriptive study design, combining a structured analysis of FDA-listed rehabilitation devices, a cross-sectional formative survey of individuals post-stroke, and targeted outreach to device manufacturers to obtain user-centered information that was consolidated into a curated dataset.

### 2.1. Device Identification and Dataset Development

We conducted a structured but iterative search to identify a sample of FDA-listed rehabilitation devices suitable for home use by individuals recovering from stroke. Our goal was to create a curated dataset of engineered devices that support independent or minimally supervised motor recovery in non-clinical environments. We focused on rehabilitation devices involving electronic sensing, actuation, stimulation, or software-based interaction, and did not aim to enumerate all passive or low-technology rehabilitation tools. This process was designed to assemble a well-defined sample of relevant devices rather than to enumerate all possible rehabilitation technologies. Rather than following a strict linear screening workflow, device identification proceeded through repeated cycles of FDA database searches, manufacturer website review, and cross-referencing with external online resources.

To identify candidate devices, we used the FDA’s *Product Code Classification* database to determine product codes relevant to stroke movement rehabilitation. We then searched the *Establishment Registration & Device Listing* database using those product codes to identify individual devices and manufacturers. Review of manufacturer websites frequently revealed additional rehabilitation devices produced by the same companies, which were then searched for in the FDA databases to determine whether they were listed and eligible for inclusion. This process led to the identification of additional relevant product codes and devices, which were subsequently queried in the FDA databases, resulting in an iterative expansion and refinement of the candidate device set and the assembly of the final device sample included in the dataset. Devices were included if their descriptions indicated that they supported stroke rehabilitation and were appropriate for use in the home. Because the FDA database does not include a standardized field indicating intended use environment, home-use suitability was determined based on explicit statements in publicly available labeling and manufacturer materials.

In addition to the FDA databases, we consulted three publicly available online catalogs of rehabilitation technologies to supplement device identification and verify product information: (1) the *NeuroRehab Directory*, which is a curated list of neurorehabilitation products that is searchable by impairment (for example, ankle/foot tightness, arm/hand weakness, balance, cognition), product category, price range, and body region; (2) the rehabilitation systems section of the *MedicalExpo* marketplace for medical equipment, which includes production-ready robotic rehabilitation systems, often with videos and marketing brochures; (3) the *Exoskeleton Report catalog*, which provides a registry of wearable exoskeletons spanning medical, industrial, military, research, and consumer applications. For all three resources, we treated entries as supplementary rather than primary inclusion sources. Devices were only added to the dataset if they could be linked to an FDA product code and met our home-use criteria. These directories frequently lacked systematic reporting of regulatory status, clinical evidence, pricing, or usability information, and are updated manually, which can lead to outdated or missing entries. As a result, we used them primarily to validate device names and manufacturers and to identify potential additional candidates for FDA code searches.

For each device, we examined manufacturer websites, online marketplaces, and available literature to extract information about device function, regulatory status, pricing, clinical validation, usability, and targeted body parts. As described in the next section, we also created fields based on information that post-stroke individuals want to know about devices. Table 1 lists the fields we chose for the dataset.

**Figure 1:**
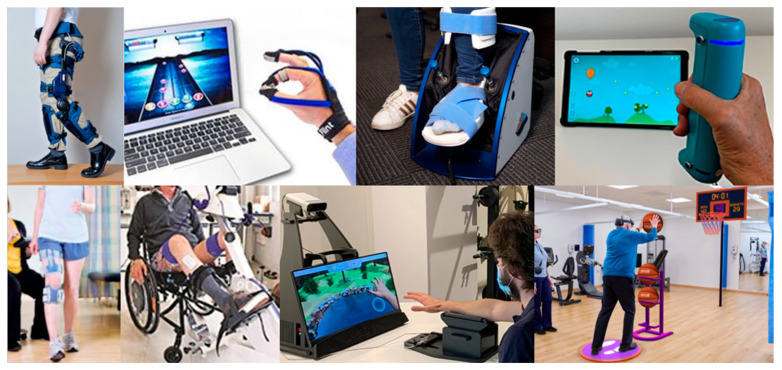
Representative examples of FDA-listed rehabilitation devices suitable for home use after stroke. The collage shows one example device for each of the eight device categories used in this study. From top left to top right: actuated wearable (*Keeogo—B-Temia, Quebec City, QC, Canada* [41]), sensorized wearable (*MusicGlove—Flint Rehab, Irvine, CA, USA* [42]), actuated robot (*Motus Foot—Motus Nova, Atlanta, GA, USA* [43], reproduced with permission from Motus Nova), and sensorized exerciser (*GripAble Home—GripAble, London, UK* [44]). From bottom left to right: wearable FES (*L300—Bioness, Valencia, CA, USA* [45]), FES bike (*RT300 FES Cycle Ergometer—Restorative Therapies, Baltimore, MD, USA* [46]), motion capture software (*RGS—Eodyne, Barcelona, Spain* [47]), and VR/AR headset (*Reality DTx—Strolll, Stafford, UK* [48]).

**Figure 2:**
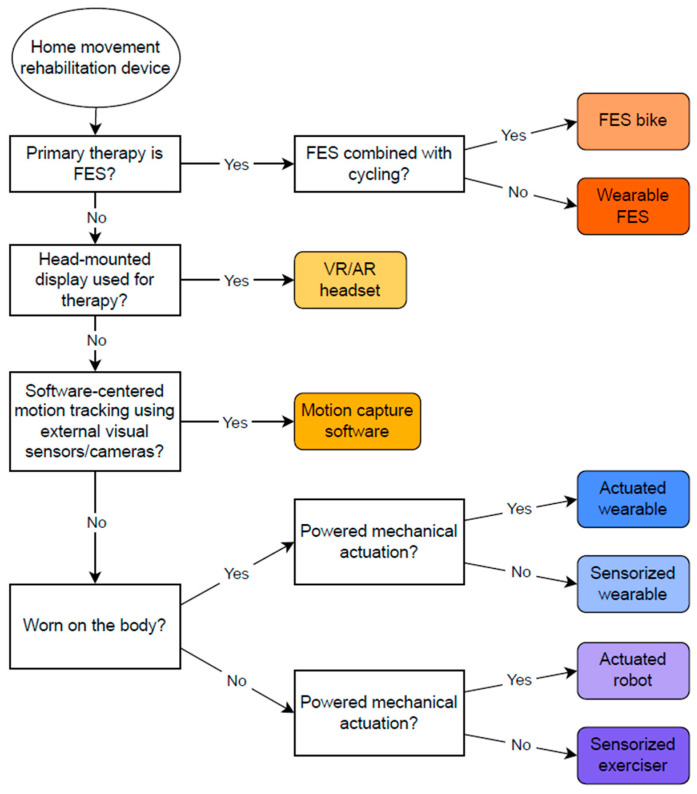
Decision tree used to classify included rehabilitation devices into eight technological categories.

We categorized all identified devices by their primary technological nature into eight groups, as summarized in Table 2 and illustrated with example devices in Figure 1. Although several prior efforts have proposed classification approaches for rehabilitation technology [38,40], existing systems are either conceptually broad or insufficiently structured to differentiate devices according to their underlying technological mechanisms and deployment characteristics. Therefore, we developed our own scheme based on explicit operational criteria reflecting the device’s primary therapeutic mechanism and deployment form factor. Actuated devices were defined by the presence of powered mechanical actuation capable of actively assisting or resisting movement, whereas sensorized devices used embedded sensors to measure movement or physiological signals in the absence of powered actuation; wearable devices were defined as those worn on the body during use, while non-wearable devices were defined as systems that interact with the user externally and are either stationary during use or handheld. Hybrid devices were classified using a hierarchical decision rule that assigns each device to a single category based on its primary therapeutic mechanism, with powered actuation taking precedence over sensing-only features. Motion capture software and Virtual Reality (VR)/Augmented Reality (AR) headset systems were classified separately as motion-sensing software technologies, distinguished by whether therapeutic interaction was delivered through external motion-tracking sensors or cameras versus a head-mounted display. Functional Electrical Stimulation (FES)-based devices were classified separately when FES was the primary therapeutic modality. Wearable FES systems were included and had their own category, while functional stimulation-integrated cycling systems were categorized separately as FES bikes. All other non-wearable electrical stimulation devices, as well as non-FES cycle ergometers, treadmill-based devices, fully passive continuous passive motion (CPM) systems, invasive systems, and systems without electronics were excluded from the dataset. In addition, devices and technologies intended solely for assessment or measurement, without a therapeutic training component, were excluded from the dataset. To improve transparency and reproducibility, this classification was implemented using a decision-tree approach, shown schematically in Figure 2.

### 2.2. Survey of Informational Needs of Individuals Post-Stroke

We designed a survey to gain insight into the types of information that stroke survivors want to know about the devices. To do this, we first conducted structured interviews of nine individuals in the chronic phase of stroke who were participating in ongoing clinical trials of rehabilitation technologies at our institution. We generated survey items by analyzing the interviews. We then administered the survey to a cohort of thirteen individuals who had a stroke. The UC Irvine Institutional Review Board approved the study and participants provided informed consent. The survey was designed as formative, hypothesis-generating work to identify important informational needs that could inform early-stage database and interface design for potential users in our regional clinical community, rather than to produce population-representative estimates of preferences among stroke survivors.

For the interview stage, a single researcher conducted all interviews, which explored participants’ recovery experiences, barriers to rehabilitation, and use of devices or apps. As described more fully in [39], we performed inductive thematic and summative content analyses on the survey transcripts, with two independent researchers coding the data and reaching consensus on possible types of informational needs that the interviewees had regarding new technologies.

From this analysis we developed a survey that asked individuals to rank the types of information they most desired when searching for new rehabilitation therapy technologies. The introduction to the survey told participants that our team was developing a website to help people find devices that might be helpful to them, and that we were interested in getting their opinions on the information the website should provide, and about how it should help them search for devices. They were told that the website would provide a basic description of each device (including how it works, what it is for, a picture, and the price), but that there are other types of information it could potentially provide. We asked them to identify their priorities for the types of information they would like the website to show from the list generated from the previous interviews, choosing first their top three highest priorities, then three medium and low priorities, followed by ranking their three highest priorities. The list of priorities they scored is presented in Table 3, Part 1.

After that, the second half of the survey told them that we wanted to get their input on how the website should convey information and asked them to rate several items from 1 to 10, with 1 being “not important” and 10 being “very important”. The list of items is presented in Table 3, Part 2.

Finally, the survey asked participants to rate how concerned they would be about privacy for a device website on a scale from 1 (not concerned) to 10 (very concerned). We also included an open-ended question in which they could describe their specific privacy concerns. Survey responses were analyzed descriptively, and mean ratings are reported to summarize relative priorities across categories, without performing inferential statistical testing. The complete survey is available in the Supplementary Materials.

### 2.3. Manufacturer Outreach

To supplement the limited information available online, we contacted the manufacturers of the identified devices directly. We emailed all the companies that manufacture the devices included in the dataset. The outreach email was written from the perspective of a researcher working closely with stroke survivors who frequently request guidance on rehabilitation devices (find the full email in the Supplementary Materials). The intent was to obtain practical, user-centered information to enable informed recommendations tailored to stroke survivors’ needs. The email requested details on four main topics:

• Pricing and Return Policy: Including device prices and whether a return policy is offered if the device does not meet user expectations or needs.
• Ease of Use: Information about how easy the device is to operate, whether it requires therapist supervision or can be used independently, any associated discomfort or pain, and the proportion of customers who use the device at home.
• Usage Requirements: Recommendations on the frequency and duration of use needed to achieve benefits, along with any scientific studies supporting the device’s effectiveness.
• Motivational Features: Descriptions of any features designed to keep users engaged, such as progress tracking, social networking, gamification, goal setting, or feedback.

The email asked companies to provide separate information for each product when multiple devices were offered. Responses were analyzed for response rate and time, responder roles, and completeness of information provided using MATLAB (R2025a, MathWorks, Natick, MA, USA).

As described in the Results, not all companies responded, so we conducted a follow-up outreach ~10 months after the initial email targeting only those that did not reply to the original email. This time, the email provided a link to a short online survey (Google Forms) designed to capture the same key user-centered information initially requested: pricing and return policy, ease of use, recommended usage patterns, and motivational features (see Supplementary Materials). The follow-up email emphasized the benefits of participation, including increased visibility of the company’s devices among the stroke recovery community. Companies with multiple devices were asked to submit a separate survey for each product to ensure accurate and specific representation. Responses to this follow-up survey were collected and analyzed alongside the original email responses, thereby supplementing the dataset and enabling us to assess both the completeness of manufacturer-provided information and companies’ overall responsiveness to user-centered inquiries.

## 3. Results

### 3.1. Characteristics of FDA-Listed Interactive Devices for Home Use After Stroke

**Figure 3:**
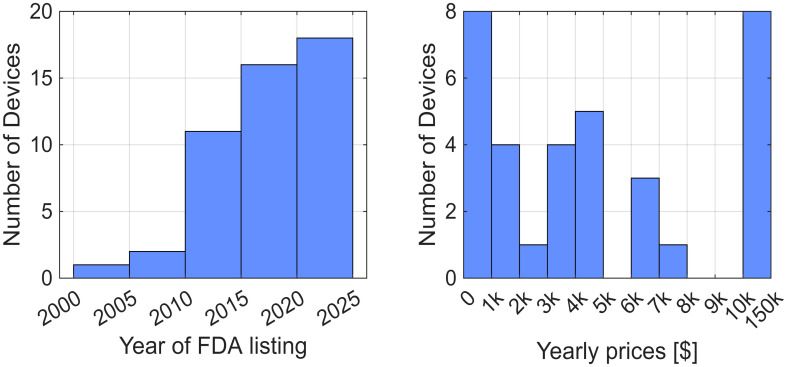
Characteristics of FDA listed rehabilitation devices suitable for home use after stroke. (**Left**) Distribution of FDA listing years for devices with available original listing dates (*n* = 48), illustrating the increasing availability of devices. (**Right**) Estimated first-year device expenses for devices with available pricing information (*n* = 34). For devices sold with a one-time purchase, the yearly cost corresponds to the upfront purchase price. For devices offered through subscription or rental models, the reported yearly cost was calculated by multiplying the stated monthly fee by twelve.

We identified 13 relevant FDA product codes (Table 4). The most frequently occurring codes were QKC (Interactive Rehabilitation Exercise Devices, Prescription Use; 26%), GZI (Stimulator, Neuromuscular; 23%), IPF (Stimulator, Muscle, Powered; 19%), and BXB (Exerciser, Powered; 9%), highlighting a strong focus on interactive exercise and neuromuscular stimulation technologies (Table 4). From these, using the FDA database, we identified 57 unique rehabilitation devices manufactured by 40 companies that met our inclusion criteria for suitability for home use by individuals recovering from stroke. Of these 57 devices, 47% were listed in the *NeuroRehab Directory*, 40% appeared in *MedicalExpo*, and 11% were included in the *Exoskeleton Report catalog*. Among the 48 devices for which listing dates were available or for which we found this information outside the FDA database, 71% were listed after 2015 (Figure 3 left). Note that listing dates were challenging to find for devices that were exempt from the 510(k) premarket notification, since the FDA database does not report listing dates for these devices.

**Figure 4:**
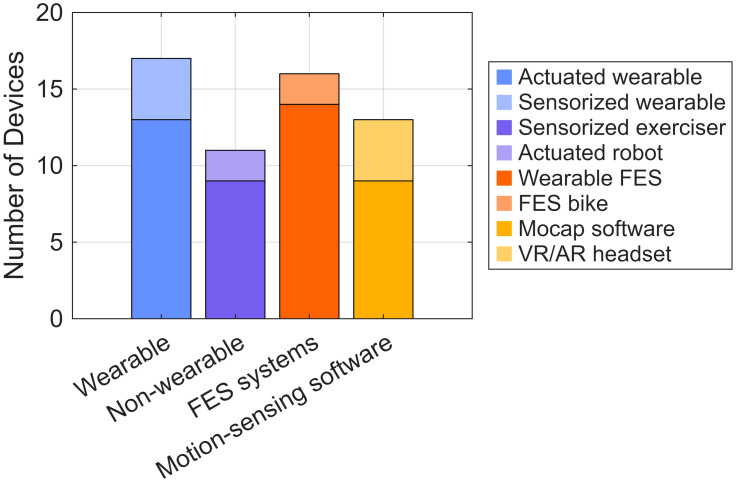
Distribution of FDA-listed rehabilitation devices suitable for home use after stroke across the eight technological categories defined in Table 2 and exemplified in Figure 1.

Device prices ranged from $60 to $130,000 (Figure 3 right). Among the 34 devices for which we obtained cost information, 8 had a yearly price under $1000 (24%), and 11 used a subscription-based pricing model (32%). With respect to body targets, 61% of devices were applicable to the hand, 49% to the ankle, 46% to the arm, 37% to the leg, and 26% to the torso. In terms of versatility, 46% of the devices were applicable to more than one joint or limb. Finally, 30% were wearables (actuated and sensorized), 19% were non-wearable training systems (actuated robots and sensorized exercisers), 28% were FES systems (wearable FES devices and FES bikes), and 23% were motion-sensing software systems (motion capture devices and VR/AR headsets) (Figure 4).

To quantify the completeness of the curated dataset and assess how much user-relevant information was publicly available online versus obtained through outreach, we summarized field-level completeness across all devices (Table 5).

### 3.2. Survey to Identify Information Priorities of Stroke Survivors

**Figure 5:**
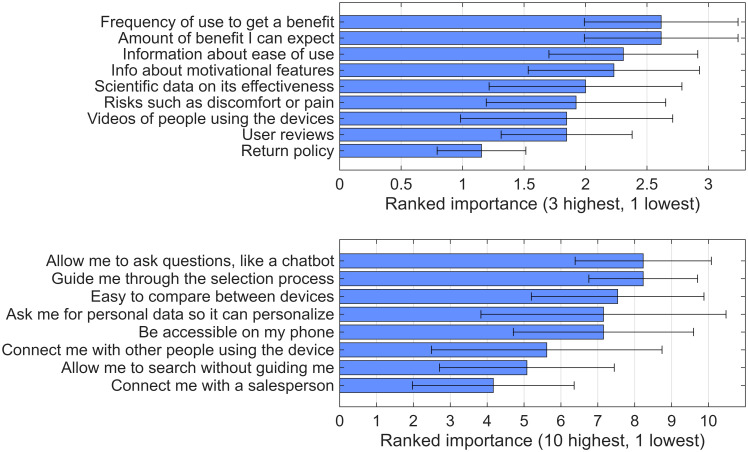
Summary of survey responses about informational needs and preferred website features. (**Top**) Participants selected their three highest priority, three medium priority, and three lowest priority types of information about rehabilitation devices from a list of nine options; for visualization, these selections were coded as high (3), medium (2), or low (1) importance, and bars show the mean importance score for each information type with error bars indicating the standard deviation. (**Bottom**) Participants rated the importance of eight possible features of a device selection website on a 10-point scale, where 1 indicated “not important” and 10 indicated “very important,” and bars show the mean rating for each feature with error bars indicating the standard deviation.

To determine which types of information about home rehabilitation technologies matter most to potential users, beyond a basic description and price, we surveyed 13 individuals with motor impairments following a stroke about their informational priorities and preferences for how a device-selection website should present this information. The results are shown in Figure 5. Participants prioritized information regarding how difficult it is to obtain benefits from these devices (including required frequency of use, ease of use, and motivational features) and how large a benefit they can expect (Figure 5 top). With respect to the website design, they prioritized a smartphone-friendly chatbot that guided users through the selection process based on their personal profile, also allowing them to compare devices (Figure 5 bottom). The lowest-rated feature was connecting to a salesperson.

With respect to the privacy question included in the survey, participants reported, on average, relatively low privacy concern for this type of website (mean 3.69 ± 3.38 on a 1 to 10 scale, where higher scores indicate greater concern). Participants’ open-ended explanations for their privacy ratings reflected several recurring themes. 33% of respondents explicitly emphasized control over personal information or identity (for example, not wanting their name or medical history shared or used for judgment), and 22% specifically referenced the need for security and Health Insurance Portability and Accountability Act protections. At the same time, 33% of participants indicated that they were willing to share information if it helped others or improved access to rehabilitation technologies, and 22% stated that privacy was not a major concern for them or was not relevant in this context.

### 3.3. Manufacturer Responses

Findings from the first half of the survey about informational needs, as reported in the previous section, were used to structure the questions in our outreach to manufacturers, ensuring we requested information on the topics most relevant to stroke survivors. We sent email inquiries to the 40 companies that manufacture the 57 devices in the dataset we created. Of these, only 13 companies (33%) responded. Nine responded within two days, and four more replied within eight days. No additional replies were received thereafter until we sent a second email 10 months later, which sought to make responding easier using an online survey. 5 companies responded to the follow-up email: three of them responded within the first 2 days, and the other two in the following 25 days, bringing the percentage of responding companies to 45%.

Among the 13 original respondents, 10 companies (77%) answered our questions directly by email, and three (23%) recommended follow-up phone conversations. We were able to schedule meetings with two of these three companies, resulting in 12 companies that ultimately provided detailed information relevant to our inquiries. The 5 companies that responded to the follow-up email completed the online survey. Overall, the 17 respondent companies supplied data for 19 devices, which corresponds to detailed information for 33% of all devices in the dataset.

**Figure 6:**
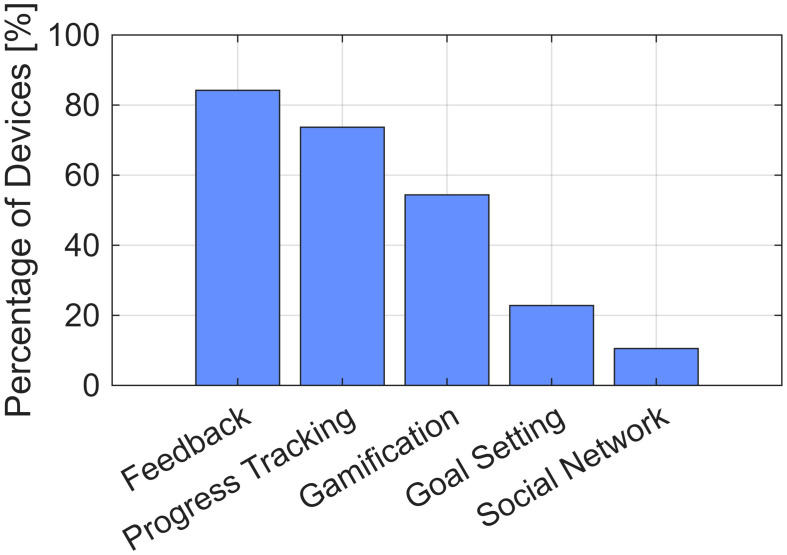
Motivational features of devices. Bars indicate the percentage of devices that include feedback, progress tracking, gamification, goal setting, or social networking functions.

16 of the 17 respondent companies supplied pricing and return policy information. 15 provided responses regarding ease of use; this information generally was qualitative, such as “Very easy to use independently” and “Quick to setup” (see Supplementary Materials). 13 described usage requirements (e.g., frequency and duration), but this was reported in widely varied form (Table 6). 14 reported on motivational features (see distribution of these features in Figure 6).

To further characterize the transparency of manufacturer responses, we conducted a structured content analysis of replies based on completeness across the ten information categories queried (price, return policy, ease of use, independent use, discomfort or pain, home suitability, frequency of use, duration of use, clinical evidence, and motivational features). For each responding company, responses were coded dichotomously to indicate whether a substantive answer was provided for each category. Across responding manufacturers (*n* = 17), 87% of queried information fields were addressed. Information on price, independent use, home suitability, and motivational features was provided by more than 94% of respondents, whereas required duration of training and the existence of supporting clinical evidence were least frequently addressed (71% and 77%, respectively). These findings indicate that although many manufacturers respond to user-centered inquiries, gaps persist in the reporting of some information that stroke survivors identified as critical for decision making.

Regarding respondent roles, 35% of the replies came from C-suite executives (e.g., Chief Executive Officer, Chief Operations Officer), 29% from sales representatives, 18% from clinical specialists, and the remaining 18% from managers, customer service staff, or engineers.

To assess potential nonresponse bias, we compared responding and non-responding manufacturers at the company level (Table 7). Response rates differed by manufacturer portfolio size: manufacturers with one or two FDA-listed devices identified in our dataset were more likely to respond than manufacturers with three or more devices. Response rates also varied by device category, with manufacturers of stimulation-based devices responding more frequently than manufacturers of wearables, non-wearable training systems, and motion-sensing software systems. Price-tier comparisons were difficult to interpret because pricing information was unavailable for many non-responding companies, limiting inference about whether response propensity differed by device cost.

## 4. Discussion

The goal of this work was to create a curated dataset of FDA-listed devices suitable for home use after stroke, and to understand which types of information matter most to stroke survivors when evaluating these technologies. This effort was motivated by the need to develop support tools that help stroke survivors and clinicians more easily identify and compare home rehabilitation technologies. We identified relevant devices through a systematic search of FDA product codes, surveyed stroke survivors about their informational needs, and contacted manufacturers to obtain missing details about price, usability, training requirements, and motivational features. The main findings were that the number of home rehabilitation devices available in the United States has expanded considerably over the last 10 years, that stroke survivors prioritize information about ease of use, training dose, expected training benefit, and motivational features, and that such information is often difficult to locate online and inconsistently reported by manufacturers. Importantly, our analysis focuses on the availability, clarity, and variability of this information across manufacturers, rather than on evaluating or comparing the actual therapeutic effectiveness of the devices themselves. Manufacturer responsiveness was limited, and recommendations for device usage as described by manufacturers lacked standardization and clear links to evidence. The information collected through this process was consolidated into the curated dataset provided in the Supplementary Materials. We discuss these results now, followed by limitations and future directions.

### 4.1. Increasing Number of FDA-Listed Interactive Devices for Home Movement Rehabilitation After Stroke

We found that, as recently as 2010, there were only a handful of devices available for home stroke movement rehabilitation listed in the FDA database; our search approach identified ~60 to analyze, and there are likely more (see below). These devices span a wide range of body targets and prices, and use a wide range of approaches, roughly balanced among wearables, non-wearables, stimulation, and motion-sensing software systems. This proliferation of devices is a positive development for stroke survivors and their caregivers. However, it also presents a practical challenge: how does one select a device? We discuss this question in the next section.

**Figure 7:**
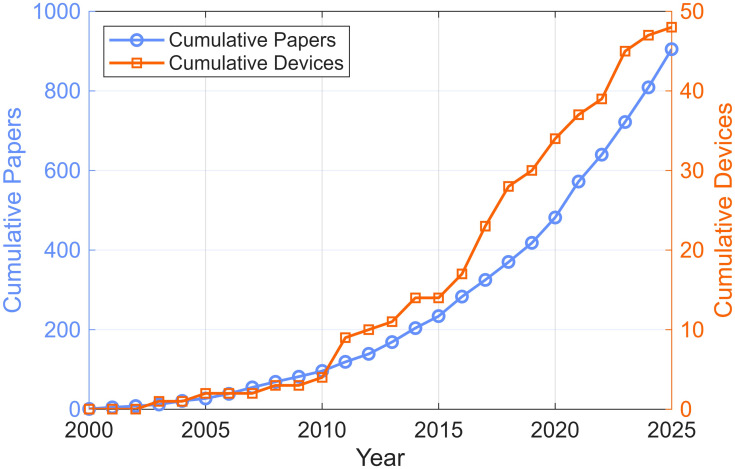
Cumulative number of publications and FDA-listed devices related to home-based movement rehabilitation after stroke. The left y-axis shows the cumulative count of PubMed-indexed papers retrieved with the query stroke [Title/Abstract] AND home [Title/Abstract] AND (robot*[Title/Abstract] OR sensor*[Title/Abstract] OR wearable*[Title/Abstract] OR stimulation [Title/Abstract] OR “motion capture” [Title/Abstract] OR “virtual reality” [Title/Abstract] OR VR [Title/Abstract] OR “augmented reality” [Title/Abstract] OR AR [Title/Abstract]) by publication year. The right y-axis shows the cumulative number of FDA-listed home rehabilitation devices identified in this study for which we found year of listing (48 devices).

Academic publications on home stroke rehabilitation technologies have also grown rapidly over the same period, at approximately the same normalized rate as FDA-listed products (Figure 7). This parallel growth suggests a linkage between academic research activity and product development. However, the number of FDA-listed devices remains only about 5% of the number of academic papers, suggesting that the majority of published work does not translate into cleared products. Future research could quantify what percentage of FDA-listed rehabilitation devices originated from inventions in academic settings, what proportion of academic papers represent clinical validation studies of FDA-listed devices, and what technical, regulatory, usability, or business factors most commonly prevent promising laboratory prototypes from progressing to FDA listing.

### 4.2. Mismatch Between User Priorities and Available Information

In terms of how people with stroke wish to select devices, survey participants consistently expressed interest in knowing how easy devices are to use without supervision, how much practice is required to obtain a therapeutic benefit, how large a therapeutic benefit they can expect, and what motivational features would support their use of the device. These priorities align with longstanding barriers to home-based rehabilitation, including uncertainty about setup, difficulty sustaining adherence, and the challenge of judging whether a device will be manageable for one’s impairment level [49,50].

A central finding of this study, however, is the gap between the information stroke survivors prioritize and what manufacturers make available to the public. Manufacturer websites and product materials rarely address usability constraints or day-to-day training expectations in clear terms. Motivational features are also described inconsistently, despite their importance for maintaining engagement. This mismatch suggests that individuals seeking home rehabilitation tools must either make decisions with partial information or rely on a clinician or researcher to interpret the scarce information available and give them recommendations. Our cross-check against existing online directories further underscored this gap, as each listed only a subset of the devices we identified and provided limited information, confirming that no single existing resource provides complete coverage of home-use stroke rehabilitation technologies and reinforcing the need for a more comprehensive, user-centered database.

The mismatch between user priorities and manufacturer disclosures likely reflects, in part, structural factors in the device listing ecosystem rather than a simple communication failure. The FDA 510(k) process evaluates devices for substantial equivalence to predicate devices, with a focus on safety and mechanical performance. It does not emphasize systematic evaluation of home usability, long-term adherence, or dose–response effects; therefore, manufacturers are not explicitly required to generate or report the kinds of information that stroke survivors value most. As manufacturers tend to generate data that satisfies regulatory requirements, user-centered information, such as ease of independent use, specific training expectations, and motivational impact, often remains underdeveloped or unpublished. When a stroke survivor asks, “Will this motivate me?”, they ask a question that the regulatory pathway does not require the manufacturer to answer.

Furthermore, the legal landscape discourages specificity regarding “Usage Requirements.” As noted in the Introduction, the dose–response relationship for movement training after stroke is complex and patient-specific (e.g., depending on the timing and location of the stroke within the nervous system). If a manufacturer were to explicitly state, “Use this device 60 min daily for 3 months to restore hand function,” they would be making a specific medical claim that requires extensive clinical evidence to substantiate. Within a 510(k) framework, this may create perceived pressure to remain conservative and non-specific about expected benefits and usage requirements. Thus, current regulatory and evidentiary expectations may contribute to the transparency gap, even as users seek more concrete guidance.

Additionally, while we did not analyze the clinical evidence for each device, this evidence is almost certainly limited, relying mostly on small studies in groups of stroke survivors who meet very specific inclusion criteria (such as minimal cognitive deficits). Enhancing the dataset with a comprehensive description of available clinical evidence is an important future direction.

### 4.3. Manufacturer Responsiveness and Transparency Gaps

The modest responsiveness of companies to direct inquiries underscores broader transparency challenges. Only a minority of manufacturers provided detailed information despite receiving multiple requests. Even among those who responded, the depth and clarity of information varied substantially, with some offering broad marketing language rather than concrete guidance. This inconsistency leaves potential users with unequal access to essential details about device operation, supporting evidence, cost structure, and return options. Improving the accessibility and completeness of device information would likely increase confidence among both users and clinicians considering technology-supported home rehabilitation.

The finding that fewer than half of manufacturers responded to inquiries, and that some responses lacked specific data, highlights the fragility of the rehabilitation technology sector. Unlike the pharmaceutical industry, which is dominated by stable giants, the rehab tech landscape is populated by small- to medium-sized enterprises and early-stage startups. A non-response to a potential customer inquiry may reflect limited resources (a third of responses came from C-suite executives), high turnover, or communication constraints. Because all devices included in the dataset were identified through the FDA Establishment Registration and Device Listing database, which requires manufacturers to register and certify their device listings annually, the associated companies were considered active at the time of data collection. Accordingly, manufacturer non-response in this study is interpreted primarily as an information-availability and transparency gap from the perspective of stroke survivors, rather than as definitive evidence of commercial inactivity.

For stroke survivors and clinicians, this introduces an additional dimension of “vendor risk”, in which a device may be technically available and FDA-listed but not actively supported or responsive to user needs. Future iterations of the dataset should therefore distinguish between devices that are only regulatorily active and those that are also commercially responsive, so that users are not guided toward options that are no longer realistically accessible or supported.

### 4.4. Challenges in Reporting Therapeutic Dose and Ease of Use

We wish to highlight a key issue revealed by this work: the lack of standardization in reporting therapeutic dose–response and ease-of-use. For dose–response, although some manufacturers provided recommended usage schedules, these guidelines were often vague or not tied to empirical data. This is consistent with broader challenges in the rehabilitation field, where dose is rarely defined with precision and lacks a consistent international reporting standard [51,52]. Furthermore, dose–response relationships remain difficult to characterize due to complex interactions between task type, duration-since-stroke, impairment level, and training context, with recent evidence suggesting that response trajectories are highly individualized [53]. A major limitation in current practice is the reliance on “time scheduled for therapy” as a dose metric, which frequently overestimates the actual active movement practice received by the patient [20,54].

The same lack of standardization appeared in descriptions of ease of use. While quantitative scales such as the System Usability Scale have recently become more common in rehabilitation robotics research, they are sporadically applied, with many studies continuing to rely on non-validated, ad-hoc questionnaires [55,56,57,58]. Furthermore, researchers have found that even when standardized scales are used, the standard benchmarks (originally developed for software and consumer electronics) do not accurately reflect the physical and cognitive complexity of rehabilitation robotics [55]. Some companies emphasized that a device is “simple” or “intuitive” without specifying the types of movements required, the need for caregiver assistance, or the physical demands on users with limited strength or dexterity. For individuals with stroke, especially those with moderate-to-severe impairments, such information is essential for determining whether a device is feasible in the home environment.

To address these issues, we propose that future work should seek to develop a standardized “Rehabilitation Device Profile” or labeling framework that could be adopted voluntarily by industry or required by purchasing organizations. This profile would translate qualitative features prioritized by users into quantifiable, comparable metrics. Although formal development would require further research and consensus building, a provisional example might include: setup burden measured as expected time to initiate active practice (minutes); an assistance index reflecting caregiver support required for setup and operation (e.g., 0–3 scale); mechanical practice intensity expressed as achievable repetitions per minute; and a cognitive interface load rating describing operational complexity.

Similarly, to improve transparency around dose–response expectations, the profile could also include, for example, standardized reporting of training dose and expected benefit, including cumulative active practice hours required to achieve a specified target outcome, an associated functional improvement index expressed as change in a clinically meaningful outcome measure, an evidential support level summarizing the strength of supporting data, and the target impairment level for which these metrics are most applicable. Together, such standardized elements would allow stroke survivors and clinicians to compare devices more meaningfully while encouraging manufacturers to align validation studies with information that directly supports real-world decision making.

### 4.5. Implications for Database Design and Clinical Decision Making

The findings directly informed the design of the stroke-focused neurotechnology dataset we include in the Supplementary Materials. Although several online catalogs of rehabilitation technologies exist, they are limited by incomplete coverage, reliance on self-listed or manually curated entries, and inconsistent reporting of regulatory status and user-relevant attributes. In particular, existing catalogs rarely provide standardized information on ease of independent use, recommended training dose, or motivational features, and they do not consistently indicate when information is missing or uncertain. In contrast, the dataset presented here is grounded in an iterative FDA-based search, refined using inclusion criteria for home use after stroke, and structured around informational priorities identified directly from stroke survivors. This dataset is currently being incorporated into a database that will serve as the knowledge base for a companion website and Large Language Model (LLM)-based chatbot interface designed to help users explore and compare rehabilitation technologies.

The idea of implementing the system as a chatbot interface was already under consideration at the onset of this work, and the survey responses reinforced this direction, as participants indicated that they would prefer an interactive, phone-accessible way to search for and compare devices rather than contacting a company salesperson. They wanted to be able to ask questions in their own words, compare devices side by side, and receive personalized information in a guided, phone-accessible process instead of feeling that they were being “sold” a specific product. Further, because stroke survivors prioritized usability, dosage guidance, and motivational elements, these items are prominent fields in the database we are developing and will be highlighted in user-facing summaries. The variability and incompleteness of manufacturers’ responses emphasize the need for clearly marking when information is verified, missing, or uncertain. Presenting device characteristics in a structured, comparable format can help users more easily navigate the increasingly crowded landscape of rehabilitation technologies and make informed decisions that align with their capabilities and goals.

Consistent with the preferences identified here, the chatbot will be designed to learn from each user’s characteristics, goals, and preferences, if granted permission to use that information. This will allow it to recommend devices tailored to their needs rather than offering generic suggestions. Confidentiality must be ensured if a user wishes to have personalized responses. At the same time, the system will present a clear disclaimer stating that the suggestions provided are for informational purposes only, do not constitute medical advice, and should not replace consultation with healthcare professionals. Users will be reminded to consult their healthcare provider, physical therapist, or occupational therapist before using any rehabilitation device, and that suitability depends on individual condition, needs, and medical history. A limitation of liability statement will also explain that the app and its Artificial Intelligence (AI) assistants are provided “as is” without warranties and that the developers are not responsible for actions taken based on the information provided. For clinicians, the database may serve as a consolidated reference that reduces the time required to review available options and supports more efficient, evidence-informed discussions with patients about home rehabilitation technologies.

### 4.6. Limitations, Future Directions, and Recommendations for Industry Standards

This study has several limitations. The survey sample size was modest and drawn from participants involved in ongoing research studies at a single institution, which may limit generalizability; accordingly, the survey findings should be interpreted as formative and hypothesis-generating, intended to inform initial database and interface design suitable to our regional clinical community. Future research should evaluate how the preferences we identified align with those of stroke populations in other geographical and socioeconomic contexts. Some devices lacked available information despite repeated outreach, leading to gaps that could not be filled without manufacturer cooperation. In addition, even though manufacturer response rates varied by company portfolio size and device category, the modest number of responding companies limits statistical inference. The analysis focused on devices listed in the United States and did not capture products available in other regulatory environments. We may also have missed some relevant FDA codes and thus excluded relevant devices. Further, we focused on devices with FDA codes that we judged to be interactive and “higher tech”, involving electronics in some ways. There are many simpler, passive tools for stroke movement rehabilitation, such as exercise bands and grip exercisers. Some passive wearable orthoses are non-actuated and non-sensorized yet mechanically support movement in clever and therapeutically useful ways. The dataset could be expanded to include such devices in the future.

An additional limitation arises from the manual and iterative nature of the device identification process. The FDA *Establishment Registration and Device Listing* database limits manual queries to a maximum of 100 devices per product code, and for four of the thirteen product codes included in this study, this limit was reached. As a result, some devices associated with these codes that met the inclusion criteria were not viewable through the database interface. Thus, the dataset analyzed in this paper should be viewed as a curated sample of FDA-listed devices.

Future work will expand the database. One possibility we are exploring is developing an AI-assisted, automated device identification pipeline using openFDA, an application programming interface (API) provided by the FDA that enables programmatic access to device registration, listing, and regulatory data. In preliminary work, using Python-based queries to the openFDA API (Python 3.13, Python Software Foundation, Beaverton, OR, USA), we have identified approximately 1260 establishment records associated with the thirteen product codes examined in this study, corresponding to more than 2000 distinct listed devices. As a feasibility test, we implemented a secondary automated screening step using LLM-assisted web searches to evaluate publicly available information for each device against predefined inclusion and exclusion criteria related to stroke rehabilitation, movement training, and home-use suitability. The LLM labeled devices that it could not confidently classify as uncertain. Manual review of devices that the LLM classified as included or uncertain led to the identification of ~40 additional devices that could potentially be incorporated into the dataset. We are currently refining and validating this approach and plan on describing it in detail in a future publication. We are hopeful that this AI-based approach will serve as the basis for a sustainable mechanism for maintaining and updating the dataset. If the dataset is to serve a public-facing, chatbot-based device recommendation system, it would be desirable if the dataset could be refreshed regularly in an automated process to reduce the risk of becoming outdated as the rehabilitation technology landscape evolves.

Additional efforts will refine data collection procedures, incorporate structured summaries of clinical evidence, and evaluate the usability and impact of the planned website and chatbot with stroke survivors, caregivers, and clinicians. Future work will also incorporate input from rehabilitation clinicians, including physiatrists and therapists, to complement survivor-identified priorities with expert perspectives on clinical efficacy, safety, and prescribing considerations.

This study suggests that the rapidly growing home rehabilitation technology field would benefit from industry-wide reporting standards. Key information should be consistently disclosed, including price, return policies, independent usability requirements, and recommended training dose to achieve a specified benefit. Motivational design elements should be described clearly enough for users to understand how the device supports sustained engagement. Aligning these reporting practices with standardized usability and dose–response metrics, as outlined above, would help transform vague claims into comparable, evidence-based summaries. Such standardization would not only help individuals and clinicians compare devices but also promote higher-quality design and clearer expectations about device capabilities, improving transparency, trust, and translation.

## Figures and Tables

**Table 1:** Dataset fields and descriptions.

Field	Description
Name	Commercial name of the device
Manufacturer	Name of the company
Website	Official site or product page
FDA Code(s)	Product classification code(s)
Year of Listing	Original year of FDA listing
Country	Manufacturer location
Price	Retail cost [$]
Price per Year	Annualized cost for subscriptions/rentals [$/year]
Trained Body Parts	Targeted body parts (e.g., hand, ankle)
Product Category	Type of product by technological nature
Return Policy	Whether a return policy is offered [Y/N]
Insurance Coverage	Whether coverage is available [Y/N]
Clinical Evidence	Existence of published validation [Y/N]
Ease of Use	Summary of independent use, accessibility, etc.
Usage Requirements	Recommended frequency and duration
Motivational Features	Features like feedback, goals, games, etc.

**Table 2:** Classification of FDA-listed home rehabilitation devices by primary technological nature.

Category	Description
Actuated wearable	Wearable device that provides active movement assistance or resistance through built-in powered actuators
Sensorized wearable	Non-actuated wearable device that uses embedded sensors to measure movement or physiological signals during task practice
Actuated robot	Non-wearable, actuated device that guides or assists limb or body movements through a powered robotic mechanism
Sensorized exerciser	Non-wearable, non-actuated device with embedded sensors that supports task-specific movement practice; treadmill- and cycle-ergometer-based systems are excluded
Wearable FES	Wearable functional electrical stimulation (FES) system that delivers neuromuscular or functional stimulation as the primary therapeutic modality, excluding stimulation-integrated cycling systems
FES bike	Stationary cycling system that combines functional electrical stimulation with a cycle ergometer for therapeutic exercise
Motion capture software	Software-centered system that uses cameras or sensors to track, quantify, and visualize body or limb movements without requiring dedicated rehabilitation hardware. Assessment-only systems are excluded
VR/AR headset	Virtual or augmented reality (VR or AR) system that delivers rehabilitation tasks through a head-mounted display

**Table 3:** Survey of informational needs’ questions.

**Part 1. Information the website should provide about the devices** (select three as most important, three as medium, and three lower)
Information about how easy each device is to use
Risks such as discomfort or pain
How often and for how long do I need to use it to get a benefit
What amount of benefit can I expect
Information about what motivational features the device offers (these could be things like progress tracking, social networks, gamification, goal setting with feedback, or motivational messages)
Videos of people using the devices
User reviews
Information about scientific studies that support its effectiveness
Return policy
**Part 2. How the website should help you find devices** (rate 1 to 10)
Ask me for personal information about my impairments and my goals so it can make personalized recommendations for what device would be best for me.
Suggest devices in a way that’s easy for me to compare them.
Allow me to ask questions like a chatbot.
Be accessible on my phone.
Guide me through the selection process.
Allow me to search without guiding me or asking me questions.
Connect me with other people who are using a device I am interested in to help me make my decision.
Connect me with a salesperson from the company that makes the device so I can get more information.

**Table 4:** FDA product codes used to identify relevant devices and percentage found.

Code	Description	Percentage
QKC	Interactive Rehabilitation Exercise Devices, Prescription Use	26%
GZI	Stimulator, Neuromuscular, External Functional	23%
IPF	Stimulator, Muscle, Powered	19%
BXB	Exerciser, Powered	9%
ION	Exerciser, Non-Measuring	7%
ISD	Exerciser, Measuring	5%
PHL	Powered Exoskeleton	5%
LXJ	Interactive Rehabilitation Exercise Devices	4%
JFA	Exerciser, Finger, Powered	4%
HCC	Device, Biofeedback	2%
PKS	Exerciser, Non-Measuring For Stroke Rehabilitation	2%
QOL	EEG-Driven Upper Extremity Powered Exerciser	2%
IQZ	Hand, External Limb Component, Powered	2%

**Table 5:** Dataset fields and completeness by source.

Field	Completeness	Found Online
Name	100%	100%
Manufacturer	100%	100%
Website	100%	100%
FDA Code(s)	100%	100%
Year of Listing	84%	72%
Country	100%	100%
Price	60%	28%
Price per Year	60%	28%
Trained Body Parts	100%	100%
Product Category	100%	100%
Return Policy	47%	16%
Insurance Coverage	42%	23%
Clinical Evidence	79%	46%
Ease of Use	61%	32%
Usage Requirements	44%	18%
Motivational Features	95%	67%

**Table 6:** Manufacturer’s response to usage requirements inquiry: “*How often and for how long would users need to use the device each week to experience benefits?*”. See dataset for further usage information obtained from the websites.

Frequency of Use
Designed to be used daily. Primarily effective while being worn; however, with regular use over a longer period of time, customers report general increases to their endurance and speed
Variable, 2–3×/week, 60 min
30 min, 7 days a week
Many chronic patients will wear the device daily for the orthotic gait benefits
Recommended to exercise at least 40 min daily
We recommend one hour per day to maximize outcomes
3 to 5 times a week 1-h sessions
3–5 times per week, up to 1 h each session
30 min, 5 days a week
Recommend a minimum of 15 min 3 times a week
Daily for as long as it is useful. Patient can wear the device all waking hours
Daily use is recommended. We recommend patients begin with one 20-min session/day and then ramp up therapy time gradually. Neuromuscular electrical stimulationtypically yields results after several months of consistent use
3 times a week for an hour
No response (4 companies out of the respondent companies, 28 companies out of the total)

**Table 7:** Company-level comparison of responding and non-responding manufacturers by portfolio size, price tier, and device category.

	Total Companies (*n*)	Responded (*n*)	Response Rate
**A. Portfolio size**			
Small (1–2 devices)	28	14	50%
Large (3+ devices)	12	3	25%
**B. Price tier**			
All devices < $5000	15	10	67%
Any device > $5000	10	7	70%
Price unavailable	15	0	0%
**C. Device category**			
Wearables	7	2	29%
Non-wearables	7	3	43%
Stimulation-based	10	7	70%
Motion-sensing software	11	3	27%
Mixed	5	2	40%

## Data Availability

The information collected for the rehabilitation devices can be found in the Dataset included in the Supplementary Materials. The survey data and manufacturer response data are available from the authors upon reasonable request.

## Supplementary Materials

The following supporting information can be downloaded at: https://www.mdpi.com/article/10.3390/bioengineering13040387/s1; Neurorehabilitation devices dataset; Survey of informational needs; Original and follow-up manufacturer outreach emails; and follow-up manufacturer outreach survey (Google Forms).

## Abbreviations

The following abbreviations are used in this manuscript:

FDA	Food and Drug Administration
FES	Functional Electrical Stimulation
VR	Virtual Reality
AR	Augmented Reality
AI	Artificial Intelligence
