# Supplementary material for: FDA-Listed Interactive Devices for Home Movement Rehabilitation After Stroke: A Mixed-Methods Study of Availability, User Needs, Information Gaps, and an Accompanying Dataset
Source: Bioengineering (Basel). 2026 Mar 27;13(4):387. doi: 10.3390/bioengineering13040387 (PMC13113761; doi:10.3390/bioengineering13040387)
Supplement: Supplementary file 1 [file bioengineering-13-00387-s001.zip › Follow-up Manufacturer Outreach Email.pdf]

Follow-up Outreach Email to Manufacturers

Dear [Company Name] team,

My name is Luis Garcia, and I'm a graduate student at **UC Irvine** working under **Professor David Reinkensmeyer**. I'm building a **database to help stroke survivors and therapists** select effective rehabilitation technologies. I reached out previously but didn't hear back, and I wanted to offer **another opportunity to be included**. This is also a **great way to increase visibility** for your devices among the stroke recovery community.

I'll provide more context below.

Warmly,
Luis

Link to the survey:
Here's the link to a short form with some quick questions. It's designed to take just a few minutes, and your input will directly inform the guidance we provide to stroke survivors so they can make informed, confident decisions. [*If the company has more than one device:* Since you offer more than one rehabilitation device, we'd really appreciate it if you could complete a separate form for each product. That way, we can make sure each one is represented accurately and fairly.]
