# Supplementary material for: FDA-Listed Interactive Devices for Home Movement Rehabilitation After Stroke: A Mixed-Methods Study of Availability, User Needs, Information Gaps, and an Accompanying Dataset
Source: Bioengineering (Basel). 2026 Mar 27;13(4):387. doi: 10.3390/bioengineering13040387 (PMC13113761; doi:10.3390/bioengineering13040387)
Supplement: Supplementary file 1 [file bioengineering-13-00387-s001.zip › Follow-up Manufacturer Outreach Survey - Google Forms.pdf]

Survey Description

We're gathering insights to map the current landscape of therapeutic and rehabilitation devices.

After interviewing 15 stroke survivors, we identified key areas where they struggle to find reliable information. This survey is your opportunity to fill that gap and ensure your technology is represented in a **public database** designed to support more informed recovery decisions.

It takes just 2–4 minutes to complete, and your contribution will directly support stroke survivors looking for trusted solutions.

Thank you for sharing your insight, it truly makes a difference.

* Indicates required question

Role Information

This information will help us better understand and interpret your responses.

1. Product Name *

2. Which department(s) best describes your role? *

Check all that apply.

- [ ] Engineering / Product Development / R&D
- [ ] Sales / Business Development
- [ ] Marketing / Communications
- [ ] Clinical Affairs
- [ ] Customer Support
- [ ] Executive Leadership
- [ ] Operations
- [ ] Other:

Pricing and Return Policy

Please answer the following questions to the best of your ability.

3. Approximately how much does this device cost? You can give an exact amount or a range. *

4. How is pricing information for the device typically communicated to potential customers? *

Mark only one oval.

- Clearly listed on the company website.
- Provided upon request through a contact form or call.
- Communicated by a direct sales representative via a quote.
- Handled by third-party distributors or clinicians.
- I'm not sure.

5. Does your company offer a return policy for its device if a user finds it does not meet their needs? *

Mark only one oval.

- Yes, we have a standard return policy (e.g., 30-90 days).
- Yes, but returns are handled on a case-by-case basis.
- No, we do not typically offer returns.
- I'm not sure.

Ease of Use & Intended Setting

This
section focuses on the usability of your company's device from the customer's
perspective.

6. What is the intended primary use setting for your company's device? *

Mark only one oval.

- Primarily for independent use by the patient at home.
- For home use, but with supervision from a caregiver.
- For home use, but with supervision from a therapist.
- For use in a clinical or hospital setting only.
- For use in both home and clinical settings.

7. How would you rate this device's ease of use for a new user? (1 = Very Easy, 5 = Very Difficult) *

Mark only one oval.

	1	2	3	4	5	
Very						Very Difficult

Usage Requirements & Efficacy

This section covers recommended use protocols and the
evidence supporting them.

8. What is your company's official recommendation for the frequency and duration of device use to achieve benefits? (e.g., "30 minutes, 5 days a week") *

9. Does your company provide scientific studies or clinical trial data to prospective customers to support the device's effectiveness? *

Mark only one oval.

- ( ) Yes, this information is publicly available on our website.
- ( ) Yes, this information is provided upon request.
- ( ) No, this information is generally not shared with customers. *Skip to question 11*
- ( ) We are in the process of gathering this data. *Skip to question 11*

Research Database Follow-up

10. Please provide link to research database, if applicable.

Motivational & Engagement Features

Please
answer these questions about features designed to promote user adherence and engagement.

11. Which of the following types of motivational features are incorporated into your company's device or its associated software/app? (Select all that apply) *

Check all that apply.

- [ ] Progress tracking (graphs, history, scores)
- [ ] Goal setting (user-defined or pre-set targets)
- [ ] Gamification (games, points, badges, leaderboards)
- [ ] Performance feedback (real-time or summary feedback)
- [ ] Social networking features (connecting with peers or therapists)
- [ ] None of the above

12. Are there new engagement or motivational features currently in development or being considered for future versions of the device? *

Mark only one oval.

- ( ) Yes
- ( ) No
- ( ) I am not aware

Contact information

If you're open to providing your name and contact information for possible follow-up, please include it below.

13. Name:

____

14. Email:

____

This content is neither created nor endorsed by Google.

Google Forms
