# Supplementary material for: FDA-Listed Interactive Devices for Home Movement Rehabilitation After Stroke: A Mixed-Methods Study of Availability, User Needs, Information Gaps, and an Accompanying Dataset
Source: Bioengineering (Basel). 2026 Mar 27;13(4):387. doi: 10.3390/bioengineering13040387 (PMC13113761; doi:10.3390/bioengineering13040387)
Supplement: Supplementary file 1 [file bioengineering-13-00387-s001.zip › Original Manufacturer Outreach Email.pdf]

Original Outreach Email to Manufacturers

Dear [Company Name] Team,

My name is Luis Garcia, and I am a PhD student working with stroke survivors as part of my research. Many of my participants ask me for recommendations on rehabilitation devices that they can use to aid in their recovery. In order to provide them with informed guidance, I am reaching out to learn more about your products.

Both my participants and I are interested in the following information:

- Pricing and Return Policy: Could you please provide details on the prices of your device(s)? Additionally, do you offer a return policy if the device doesn't meet expectations or specific needs?
- Ease of Use: How easy is it to use the device? For example, would a therapist need to be present, or could it be used independently? Is there any potential discomfort or pain associated with its use? Also, what percentage of your customers use the device at home?
- Usage Requirements: How often and for how long would users need to use the device each week to experience benefits? Are there any scientific studies or papers that back up its effectiveness?
- Motivational Features: Do your devices offer features to keep users engaged, such as progress tracking, social networks, gamification, goal setting, or feedback?

If your company offers more than one device, I would appreciate independent information for each product to ensure that I can make specific recommendations tailored to the needs of my participants.

Your responses will greatly assist me in helping stroke survivors make informed decisions about rehabilitation tools. Any additional information you can provide is much appreciated.
Thank you for your time, and I look forward to your response.

Sincerely,
Luis
