# Supplementary material for: FDA-Listed Interactive Devices for Home Movement Rehabilitation After Stroke: A Mixed-Methods Study of Availability, User Needs, Information Gaps, and an Accompanying Dataset
Source: Bioengineering (Basel). 2026 Mar 27;13(4):387. doi: 10.3390/bioengineering13040387 (PMC13113761; doi:10.3390/bioengineering13040387)
Supplement: Supplementary file 1 [file bioengineering-13-00387-s001.zip › Survey of Informational Needs.pdf]

Survey of Informational Needs

Companies have made a lot of new devices to help people who have had a stroke continue their rehabilitation therapy and exercise at home. These are things like computer games, wearable sensors, exoskeletons, robotic devices, exercise devices, virtual reality, and electrical stimulation There are dozens of these devices now, but they are hard for people to find and compare. We are developing a website to help people find devices that might be helpful for them. We are interested in getting your opinion on what information this website should provide to you, and also about how it should help you search for devices.

Part 1: Information it should provide about the devices. The website will provide a basic description of each device (including how it works, what it's for, a picture, and the price). But there are other types of information it could potentially provide. Please identify your priorities for the types of information you would like it to show you from the following list. Please choose the 3 that are most important to you, and label them with an "H" (High), 3 that are of medium importance and label them with an "M" (Medium), and 3 of lower importance and label them with an "L" (Low). If there is anything missing from this list that you think should be on it, please let us know and we can add it.

[ ] 1. Information about how easy each device is to use.

[ ] 2. Risks such as discomfort or pain

[ ] 3. How often and for how long do I need to use it to get a benefit?

[ ] 4. Amount of benefit I can expect?

[ ] 5. Information about what motivational features it offers. (These could be things like progress tracking, social networks, gamification, goal setting with feedback, or motivational messages)

[ ] 6. Videos of people using the devices

[ ] 7. User reviews

[ ] 8. Information about scientific studies that support its effectiveness

[ ] 9. Return policy

[ ] 10. Other: ____________________

For the 3 things you selected as most important (H), can you rank them below in order of most important (1), to least important (3) by putting the number next to the ranking below ?

1. [ ]
2. [ ]
3. [ ]

Comments:

Part 2: How the website should help you find devices: OK, now in this second part of our survey, we want to get your input on how the website should work. Again, remember, this is a website that will contain a database of devices with a description of each device (including how it works, what it's for, picture, price, as well as the information you identified above). But now, we want to understand how you would like to be able to search through the website. For this question, please rate each item in the below list from 1 to 10, 1 being "not important" and 10 being "very important".

The website should:

[ ] 1. Ask me for personal information about my impairments and my goals so it can make personalized recommendations for what device would be best for me

[ ] 2. Suggest devices in a way that's easy for me to compare them

[ ] 3. Allow me to ask questions, like a chatbot

[ ] 4. Be accessible on my phone

[ ] 5. Guide me through the selection process

[ ] 6. Allow me to search without guiding me or asking me questions

[ ] 7. Connect me with other people who are using a device I am interested in to help me make my decision

[ ] 8. Connect me with a salesperson from the company that makes the device so I get more information.

Follow-up questions:

How concerned are you about privacy for a website like this?
1: Not concerned, 10: very concerned

Why or why not?
